# Human umbilical cord mesenchymal stem cells combined with porcine small intestinal submucosa promote the healing of full-thickness skin injury in SD rats

**DOI:** 10.2144/fsoa-2023-0123

**Published:** 2024-05-20

**Authors:** Xu XiaoMing, Chen Yan, Gu JiaMing, Liang LiTao, Zhang LiJuan, Song Ying, Yuan Lu, Song Qian, Dong Jian

**Affiliations:** 1 Yunnan Tumor Research Institute, The Third Affiliated Hospital of Kunming Medical University, Yunnan Provincial Tumor Hospital/Yunnan Cellular Therapy & Quality Control System Engineering Research Center, Kunming, Yunnan, 650118, China; 2 Department of Pathology, The Third Affiliated Hospital of Kunming Medical University, Yunnan Provincial Tumor Hospital, Kunming, Yunnan, 650118, China; 3 Department of Obstetrics, The Second Affiliated Hospital of Kunming Medical University,Kunming,Yunnan, 650101, China; 4 Department of Obstetrics, Kunming Maternal & Child Health Hospital, Kunming, Yunnan, 650011, China

**Keywords:** full-thickness skin injury, human umbilical cord mesenchymal stem cells combined with porcine intestinal submucosa, SD rats, vascularization

## Abstract

**Aim:** To assess the therapeutic potential of human umbilical cord mesenchymal stem cells (hUCMSCs) combined with porcine small intestinal submucosa (SIS) on full-thickness skin injuries in rats. **Methods:** We established full-thickness skin injury models in Sprague–Dawley rats, dividing them into blank control, SIS, hUCMSCs and hUCMSCs combined with SIS. We monitored wound healing, scores and area, and analyzed inflammatory cells, microvessel density and collagen fibers after 12 days. **Results:** The blank group showed no healing, forming a scar of 0.6 × 0.5 cm^2^, while SIS and hUCMSCs groups exhibited incomplete healing with 0.4 × 0.5 cm^2^ scabs. Wound healing was significantly better in the hUCMSCs combined with the SIS group. **Conclusion:** Local application of hUCMSCs combined with SIS enhances full-thickness skin injury wound healing in rats.

## Plain language summary

Our skin protects us from infections and injuries, but severe damage can lead to health problems. In this study, we explored a promising new treatment to enhance skin healing. We used mesenchymal stem cells derived from umbilical cords in combination with a biological material called porcine small intestinal submucosa (SIS) to conduct experiemnts on rats with skin wounds. This treatment led to much better healing in rats with deep skin wounds compared with standard approaches. This approach is promising for treating severe skin injuries, offering hope for quicker recovery and better outcome, including faster recovery, reduced pain and inflammation and less scarring.

Wound repair of full-thickness skin lesions caused by severe trauma, burns, and chronic diseases has become a serious problem worldwide. Traditional drug therapy and surgical treatment are mainly used, and new cell therapy methods are also applied to clinical patients [1]. Drug treatment takes a long time and takes effect slowly, which seriously affects the quality of life of the subjects. For severe skin damage, surgical treatment includes surgical excision of diseased tissue and repair with autologous skin grafting, allogeneic skin grafting, and other repair materials. However, both autologous and allogeneic skin grafts are limited by donor origin and immune rejection. In addition, reconstructive surgery may not be successful, or the wound may be broken again at the original site several years after healing [2,3].

Cell therapy clinical results show that mesenchymal stem cells have the epidermis cell differentiation potential, can promote the healing of damaged skin, ecto mesenchymal stem cells will apply to the skin burn, diabetic foot ulcers, radioactivity skin damage does not heal the wound, can control the progress of the disease, promote the wound repair, achieved good therapeutic effect [4]. However, cell therapy is not ideal for repairing full-thickness skin injury, and it is difficult to repair it and restore its normal physiological function effectively. Stem cells in the treatment of full-thickness skin tissue damage effect is limited, and the existing fiber structure of stem cells can not simulate the extracellular matrix, and stem cell secretion of cytokines and bioactive ingredients can promote skin epidermal cells and epithelial cell growth in structure, can't repair full-thickness skin damage, the lack of essential amino acid and collagen synthesis of muscle fiber raw materials. It is urgent to find a new treatment strategy to fundamentally solve the difficulty of healing full-thickness skin injuries [5,6].

Mesenchymal stem cells (MSCs) are classified as adult stem cells due to their capacity for regeneration and ability to differentiate into multiple cell lineages. MSCs possess a broad range of potential uses, either in their biological state or when combined with certain biomaterials to generate nanocomposites. Natural or synthetic biomaterials are currently employed in utilizing metallic and non-metallic nanoparticles (NPs) to encapsulate MSCs within hydrogels such as alginate or chitosan and load drug cargo into MSCs. On the other hand, nanofibers derived from various polymer scaffolds, including polycaprolactone (PCL), poly-lactic-co-glycolic acid (PLGA), poly-L-lactic acid (PLLA), silk fibroin, collagen, chitosan, alginate, hyaluronic acid (HA), and cellulose, are employed to directly support or facilitate the growth of MSCs on these scaffolds. The utilization of MSCs-based nanotherapies demonstrates their potential in several fields of biomedicine, including wound healing, bone and cartilage engineering, cardiac problems and neurological illnesses [^7-9^]. MSCs, characterized by their self-renewal capacity and ability to differentiate into various cell lineages, have been harnessed for their unique regenerative properties. These cells have shown promising results in numerous preclinical and clinical studies, fostering the development of cutting-edge therapies for various medical conditions, including tissue injuries and degenerative diseases. MSCs' immunomodulatory and paracrine effects have been attributed to their success in promoting tissue repair and modulating the local microenvironment, ultimately enhancing the body's innate regenerative mechanisms [^10-12^]. In the context of wound healing and tissue regeneration, various studies have explored the potential of MSC-based biomaterials to optimize these cells' therapeutic benefits [13]. These biomaterials often serve as a scaffold, providing structural support and creating an environment conducive to cellular adherence, proliferation and differentiation. Incorporating MSCs within such biomaterials enhances their regenerative potential, allowing for more controlled and efficient healing processes [14].

Biomaterials have high elasticity and large strain and have been used in various engineering applications of soft tissues, including muscle, vocal cords, bladder, skin and adipose tissue [^15-17^]. As a natural material, the small intestinal submucosa (SIS), an acellular matrix material, has special biological activities to regulate the growth and development of cells and does not easily cause immune rejection [18]. However, applying SIS combined with human umbilical cord mesenchymal stem cell material in full-thickness skin injury and exploring its biological functional activity remain largely unknown. This study aimed to investigate the effect of SIS combined with human umbilical cord MSCs on treating full-thickness skin injury in rats. In light of these advancements, our study investigates the synergistic effects of human umbilical cord-derived MSCs and SIS, an ECM-based biomaterial, to accelerate skin injury healing in SD rats. By combining the regenerative potential of MSCs with the supportive framework of SIS, we aim to provide new insights into optimizing wound healing strategies.

## Materials & methods

### Design randomized controlled animal experimental study

#### Time & Place

The experiment was completed at the Cancer Institute of Yunnan Cancer Hospital from April to May 2022.

### Material

Experimental animals: 35 3-month-old healthy female Sprague–Dawley (SD) rats (body weight 180–210 g) were provided by the Animal Experimental Department of Kunming Medical University. The treatment of rats during the experiment followed the requirements of the Experimental Animal Ethics Committee of Kunming Medical University.

Experimental materials: porcine small intestinal submucosa and stem cell preparation. Beijing Bohuizhujin Biotechnology Co., LTD, produces porcine small intestinal submucosa. The stem cell preparation included human umbilical cord mesenchymal stem cells. The human umbilical cord mesenchymal stem cells used in this study contained 1 × 10^6^ human umbilical cord mesenchymal stem cells in 1 ml of stem cell preparation.

### Experimental methods

Experimental animal grouping: 35 SD rats were randomly divided into blank control group, porcine small intestinal submucosa group, human umbilical cord mesenchymal stem cells group, and human umbilical cord mesenchymal stem cells combined with porcine small intestinal submucosa group, with five rats in blank control group and ten rats in each other group. Before the experiment on dorsal skin, the hair was shaved using electrical clippers and was anesthetized by intraperitoneal injection of 3% sodium pentobarbital. Each group received skin wounds on the buttocks and back, and the wound area was 1 × 1 cm^2^, deep to the muscle layer.

The following interventions were given on the second day: negative control group, no treatment. Five pieces of 0.25 × 0.25cm^2^ SIS material were evenly applied to the wound in the porcine small intestinal submucosa group. Human umbilical cord mesenchymal stem cells group: (1 × 10^6^ cells, 1.2 g according to the wound area of 15 cm^2^). The wound was evenly treated with a suspension containing 1 × 10^5^ mesenchymal stem cells, 0.1 g, once a day for 6 days. Human umbilical cord mesenchymal stem cells combined with porcine small intestinal submucosa treatment group: (1 × 10^6^ cells, 1.2 g according to the wound area of 15 cm^2^), wound uniform application size of 0.25 × 0.25 cm^2^, containing 1 × 10^5^ mesenchymal stem cells SIS complex, 0.1 g, once a day, for 6 days.

On the third day of composite culture, the porcine small intestinal submucosa composite human umbilical cord mesenchymal stem cell material was fixed with 2.5% glutaraldehyde, dehydrated with gradient acetone, replaced with isoamyl acetate, and dried to the critical point. Then, the surface was sprayed with gold, and the adhesion, growth, division and proliferation of human umbilical cord mesenchymal stem cells in porcine small intestinal submucosa were observed by scanning electron microscopy.

Wound healing and wound area 1) general observation of skin wounds: from the first use, the healing of skin wounds of rats in each group was recorded every 2 days and observed by taking photos for 12 days. 2) After treatment, the skin wound area of the rats in each group was measured once every 2 days: paste the sterilized transparent patch on the wound surface, draw a line on the wound surface and use a graphic analyzer to process the graphic area.

Observation of wound histopathological morphology on the 12th day of treatment: new granulation and skin tissue of the wound were cut and placed into a centrifuge tube containing 10% formaldehyde fixation solution. The Department of Pathology performed Hematoxylin and eosin staining and then observed under a microscope. Observation of wound collagen fibers on the 12th day of treatment: wound tissues were put into a centrifuge tube containing 4% formaldehyde fixation solution, and the Department of Pathology performed Masson staining to observe the arrangement and morphology of wound tissues with a light microscope and the images were recorded. As reported previously by Blanco *et al.* [19], the observation of wound microvessel density hematoxylin and eosin-stained sections were stained with CD31 antibody, and ten non-overlapping visual fields were randomly selected for observation at 200 magnification, and wound microvessels were counted, and their mean values were counted. The ImageJ software was used to quantify the expression of hUC-MSC markers within the tissue sections.

The main observational indicators are differences in wound healing, wound area, microscopic inflammatory response, microvessel density, tissue collagen arrangement and morphology of rats.

### Statistical analysis

The statistical software GraphPad Prism 6.0 was utilized for data processing, and the data were expressed as the mean ± standard deviation. The statistical method employed to compare groups was the one-way analysis of variance (ANOVA), with a significance level of p < 0.05, indicating a statistically significant difference.

## Results

### Culture of human umbilical cord mesenchymal stem cells, expression markers detected by flow cytometry & scanning electron microscopy

**Figure 1.**
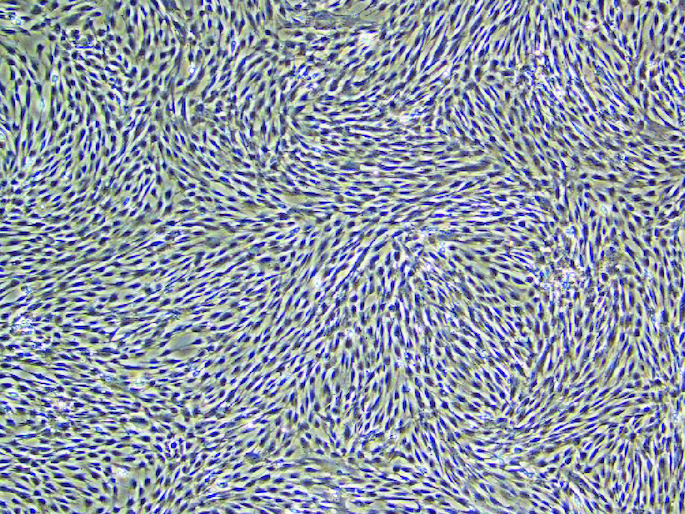
In a 400-fold magnified photo of human umbilical cord mesenchymal stem cells under an inverted microscope.

Culture of human umbilical cord mesenchymal stem cells (Figure 1)

**Figure 2.**
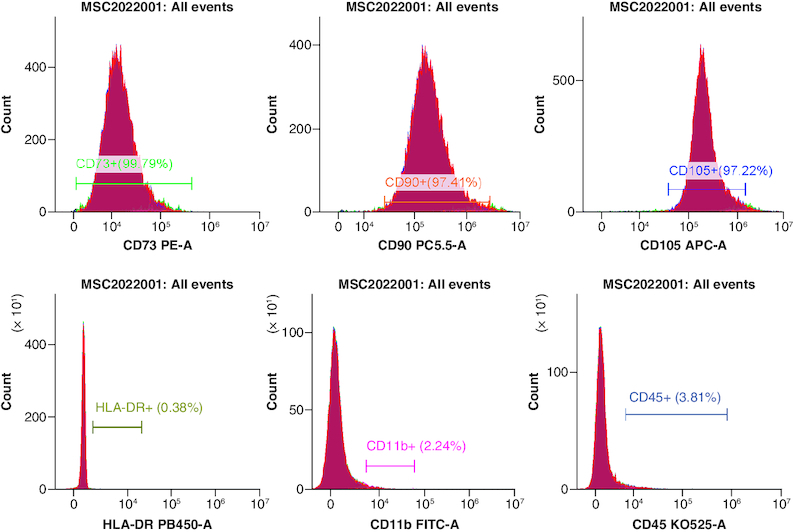
Positive indicators CD73^+^, CD90^+^ and CD105^+^ are all greater than 95.0%, while the negative indicators HLA-DR+, CD11^+^ and CD45^+^ are all less than 2%, meeting the standard.

The effectiveness of flow cytometry in detecting human umbilical cord mesenchymal stem cells (cell phenotype identification) (Figure 2).

**Figure 3.**
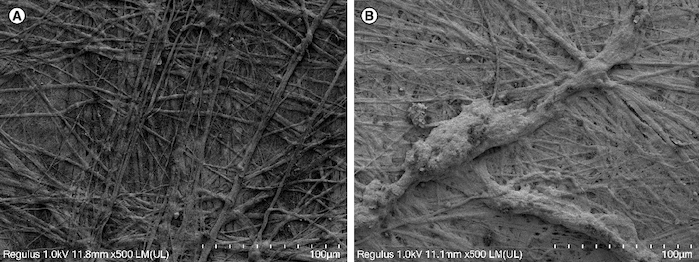
Electron microscopy. **(A)** Small porcine intestinal submucosa. **(B)** Human umbilical cord mesenchymal stem cells combined with small porcine intestinal submucosa (scale bar = 100 μm).

Scanning electron microscope observations of the composite materials are shown in Figure 3 below.

### Wound healing, wound score & wound area

#### General observation

**Figure 4.**
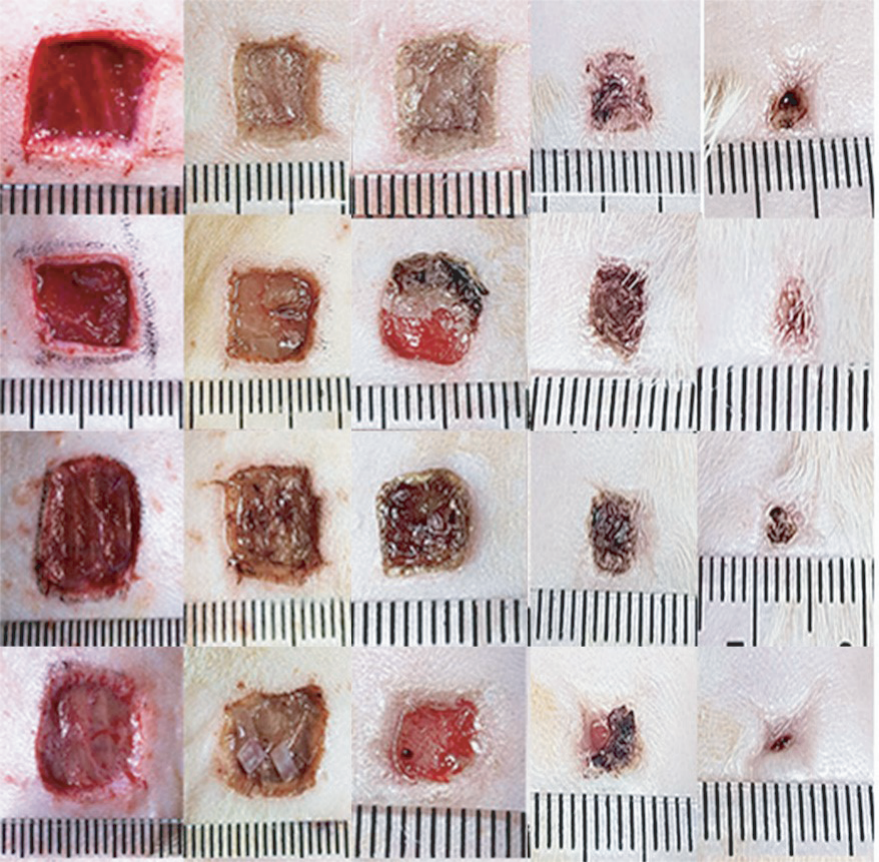
The effect of human umbilical cord mesenchymal stem cells combined with porcine small intestinal submucosa on wound healing.

In the blank control group, the skin wound did not heal during the observation period and the size of brown scar tissue was about 0.6 × 0.5 cm^2^. In the small intestinal submucosa group, the skin wound healing was incomplete and the size of scab scar tissue was 0.4 × 0.5 cm^2^. In the human umbilical cord mesenchymal stem cell group, the skin wound healing was incomplete and the size of scab scar tissue was 0.4 × 0.4 cm^2^. The human umbilical cord mesenchymal stem cells combined with porcine small intestinal submucosa grew the new epithelial tissue, and the wound healed completely during the observation period (Figure 4 & Table 1).

**Table 1.** Wound area after treatment (cm^2^), (±s, blank control group n = 5, small porcine intestinal submucosa group, human umbilical cord mesenchymal stem cell group, and human umbilical cord mesenchymal stem cells combined with small porcine intestinal submucosa group. n = 10).

Group	0 Day	3 Day	6 Day	9 Day	12 Day
Blank control group	1.32 ± 0.56	1.31 ± 0.78	1.29 ± 0.64	1.09 ± 0.89	0.68 ± 0.81
Small intestinal submucosa group	1.36 ± 0.42	1.35 ± 0.81	1.33 ± 0.83	0.88 ± 0.76	0.43 ± 0.42
Human umbilical cord mesenchymal stem cell group	1.39 ± 0.71	1.38 ± 0.62	1.27 ± 0.65	0.83 ± 0.42	0.38 ± 0.58
Human umbilical cord mesenchymal stem cells combined with porcine small intestinal submucosa	1.44 ± 0.37	1.43 ± 0.76	1.15 ± 0.69	0.57 ± 0.66	0.16 ± 0.25

After statistical analysis, the wound area of human umbilical cord mesenchymal stem cells combined with the porcine small intestinal submucosa group was compared with the blank control group, porcine small intestinal submucosa group, and human umbilical cord mesenchymal stem cell group at 9 and 12 days after treatment, p < 0.05.

### Observation of inflammatory reaction by paraffin section

**Figure 5.**
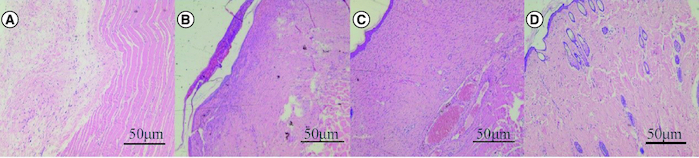
Effect of human umbilical cord mesenchymal stem cells combined with small porcine intestinal submucosa on inflammatory cells (H&E staining ×200). **(A)** Blank control group. **(B)** Small porcine intestinal submucosa group. **(C)** Human umbilical cord mesenchymal stem cell group. **(D)** Human umbilical cord mesenchymal stem cells combined with small porcine intestinal submucosa group (scale bar = 50 μm).

The blank control group had no epidermal structure under the microscope, but more inflammatory cells infiltrated. The SIS group had obvious inflammatory cell infiltration, reaction and exudation. Human umbilical cord mesenchymal stem cells showed inflammatory cell infiltration, less ulcer surface and better healing. Human umbilical cord mesenchymal stem cells combined with the SIS group showed good healing of skin lesions, growth of skin appendages and hair follicles (Figure 5).

The number of inflammatory cells in the blank control group was 48 ± 8.9/visual field, the number of inflammatory cells in the porcine small intestinal submucosa group was 35 ± 6.3/visual field, the number of inflammatory cells in the human umbilical cord mesenchymal stem cell group was 29 ± 5.8/visual field, and the number of inflammatory cells in the human umbilical cord mesenchymal stem cell combined with SIS group was 12 ± 4.5/visual field. There was a significant difference between the human umbilical cord mesenchymal stem cell complex SIS group and the other three groups (p < 0.05).

### Observation of wound microvessel density (CD31 staining)

**Figure 6.**
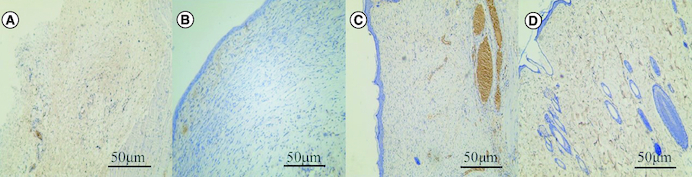
Effect of human umbilical cord mesenchymal stem cells combined with small porcine intestinal submucosa on wound microvessel density (CD31, ×200 magnification). **(A)** Blank control group. **(B)** Small porcine intestinal submucosa group. **(C)** Human umbilical cord mesenchymal stem cell group. **(D)** Human umbilical cord mesenchymal stem cells combined with small porcine intestinal submucosa group (scale bar = 50 μm).

There were few microvessels in the blank control group. In the SIS group, few capillaries were confined to the superficial dermis. There were more blood vessels in human umbilical cord mesenchymal stem cells. Human umbilical cord mesenchymal stem cells combined with SIS showed abundant blood vessels, similar to normal skin (Figure 6).

The positive rate of CD31 cells was 2.69 ± 6.1/ field in the blank control group, 10.5 ± 3.4/field in porcine small intestinal submucosa group, 12.7 ± 3.5/field in human umbilical cord mesenchymal stem cells group, and 23.7 ± 5.4/field in human umbilical cord mesenchymal stem cells combined with porcine small intestinal submucosa group. The positive expression rate of CD31 in microvessel density of human umbilical cord mesenchymal stem cells combined with the porcine small intestinal submucosa group was the highest, and the difference was statistically significant compared with the other three groups (p < 0.05).

### The results of collagen fiber staining in wound tissue

**Figure 7.**
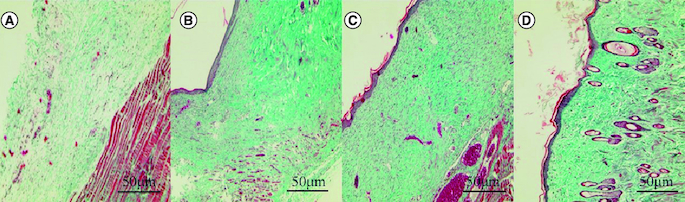
Effect of human umbilical cord mesenchymal stem cells combined with small porcine intestinal submucosa on wound collagen fibers (Masson staining, ×200). **(A)** Blank control group. **(B)** Small porcine intestinal submucosa group. **(C)** Human umbilical cord mesenchymal stem cell group. **(D)** Human umbilical cord mesenchymal stem cells combined with small porcine intestinal submucosa group.

In the blank control group, tissue edema, loose collagen fiber structure and obvious fracture were observed. In the SIS group, collagen fibers proliferated and arranged parallel to the epidermis, which was relatively immature. Immature collagen fibers were found in the human umbilical cord mesenchymal stem cells group, more collagen fibers were found in the epidermal layer and fiber proliferation was more than that in the SIS group. The collagen fibers of human umbilical cord mesenchymal stem cells combined with the SIS group were thick, dense and orderly. The collagen fibers in the wound of human umbilical cord mesenchymal stem cells combined with the SIS group were more orderly than those of the other three groups (Figure 7).

## Discussion

The skin is the body's largest organ and acts as a protective barrier that minimizes the invasion of viruses and bacteria. Skin lesions such as burns and chronic wounds often cause severe pain, rapid and dangerous water loss, and bacterial infections that affect internal homeostasis and can even threaten a person's life. As a result, skin wounds, especially large and full-thickness wounds, can seriously impact human life and health. Given these risks, appropriate full-thickness wound dressings are urgently needed [4,20].

Cell therapy is a novel and promising tool for tissue regeneration [^21-26^]. Stem cell therapy is a method to accelerate the angiogenesis of the ischemic tissues [4,27]. Stem cells can differentiate into multiple lineages and secrete various growth factors, and/or the potential of cytokines for treating ischemic tissues plays a beneficial role [^28-31^]. A study reported that the accumulation of cells can enhance the survival rate of flaps between bone marrow mesenchymal stem cell groups. This effect may be related to promoting angiogenesis and reducing inflammation. The extraction and secretion of growth factors have been reported as one of the important functions of bone marrow mesenchymal stem cells [31,32]. The regenerative role of bone marrow mesenchymal stem cells in the skin has been confirmed in the literature [33].

Bone marrow mesenchymal stem cells can accelerate wound healing by secreting a large amount of vascular endothelial growth factor A. Studies have shown that the administration of bone marrow mesenchymal stem cells at the wound site can increase the level of specific factors on the 7th day and help wound healing [34]. Vascular endothelial growth factor is essential for endothelial cell migration [35] and proliferation [36]. As cytokines increase, more inflammation occurs in the wound area. After the occurrence of inflammation, bone marrow mesenchymal stem cells exert their immunomodulatory properties and affect the wound surface [37]. An earlier study had shown the extent of cell loss during or after transplantation at the wound site.

However, traditional stem cell drug delivery methods, whether spray or local injection, have many challenges and problems due to the heterogeneity of cell suspension and the lack of control over the injection site [38]. Most clinical trials have used the injection of suspensions of therapeutic cells, including mesenchymal stem cells, in liquid carrier solutions, many of which result in poor cell retention and reduced therapeutic efficacy [39]. To overcome this problem, efforts are being made to find natural materials that can improve stromal cell survival and have therapeutic potential [21,40].

The purpose of engineering tissue scaffold material is to provide a 3D scaffold for constructing tissue cells, which is conducive to cell adhesion, proliferation, and differentiation, and to provide a good external environment for cell growth. By supporting, the tissue maintains its shape. The template function provides a place for cells to attach, grow, differentiate, increase, and metabolize, guide the regeneration of damaged tissue, and reshape the structure of regenerative tissue. Currently, most choices are synthetic degradable polymer materials, such as polylactic acid (PLA) and polyglycolic acid (PGA). However, the study found that this synthetic biomaterial has no biological activity, lacks cell recognition receptors, has poor surface hydrophobicity, and has poor cell adhesion, which all affect cell adhesion and interaction on the scaffold.

The small intestinal submucosa (SIS) is an acellular, collagen-rich extracellular matrix material derived from the porcine small intestinal submucosa [41,42]. SIS comprises collagen types I, III, V and VI and a small amount of collagen type IV. It also contains a variety of cytokines, such as VEGF, bFGF, TGF-B, EGF, chondroitin sulfate, glycosaminoglycan, fibronectin, heparin, heparan sulfate and hyaluronic acid [43]. These signals help cell proliferation, blood vessel formation and differentiation.

SIS is widely used in clinical practice for tissue remodeling, especially for structural support [44]. SIS has many advantages over other types of extracellular matrix. SIS's porous, 3D structure allows cell adhesion, proliferation and differentiation [^45-47^]. In addition, SIS is an excellent scaffold for reconstructing damaged tissue. Because of its low immunogenicity, it does not cause immune-mediated inflammation after transplantation. It plays a crucial role in skin regeneration. Ahn *et al.* transplanted bone marrow mesenchymal stem cells onto SIS and cultured *in vitro*. The results showed that BMSCS had good adhesion and proliferation ability on the SIS surface. Human umbilical cord mesenchymal stem cells have multidirectional differentiation potential, high proliferation ability, easy extraction, non-invasive collection, good histocompatibility, and low immune rejection [48], and are considered ideal seed cells for tissue engineering.

In this study, we designed a novel and lightweight SIS composite. We used porcine small intestinal submucosa (SIS), an acellular SIS matrix composite, as a natural material with special biological activity, regulates cell growth and development and does not easily cause immune rejection. Combined with human umbilical cord mesenchymal stem cells as an implantable scaffold for tissue repair, it can repair full-thickness skin injuries and accelerate wound healing in rats. The results showed that the SIS bilayer could promote the proliferation and migration of wound cells and accelerate wound healing. This result can be explained by covering the wound with the SIS bilayer creates a suitable moist microenvironment [49].

Researchers believe that overcoming vascular dysregulation is one of the most basic treatment options for wound healing, and diabetic ulcers and vascular diseases have similar challenges [50]. Several studies have shown that inducing more blood flow at these wound sites can improve the repair process [51]. The increase in vessel density after BMSCS administration results from the metabolic needs of ischemic sites [52]. However, the distribution of blood vessels in the skin is extremely uneven, and the overall position of capillaries cannot be represented by 2D information such as surface thickness. This study uses small intestinal submucosa of plasticity after water, and the small intestine submucosa scaffold of a 3D culture system can load all kinds of growth factors, provide an adhesive matrix for transplanted cells, promote cell proliferation and differentiation, and accelerate the process for transplant carrier revascularization, promote human umbilical cord mesenchymal stem cells can better adapt to damage between organizations, By giving full play to its better biological characteristics, the wound can benefit from 2D information transmission to 3D repair effect, to introduce new stereological methods. Human umbilical cord mesenchymal stem cells combined with porcine small intestinal submucosa can achieve real three-dimensional morphological structures to repair full-thickness skin injury wounds [53].

CD31 is a marker of blood vessels and angiogenesis at the wound site. In this study, immunohistochemistry showed that the expression of CD31 in human umbilical cord mesenchymal stem cells combined with the SIS group was significantly higher than in other groups on day 12, and the blood vessels were abundant, similar to normal skin.

Some studies have reported that BMSCS implanted into the small intestinal submucosa can ameliorate bile duct injury in rabbits, and BMSCS with SIS have increased growth compared with BMSCS alone. HE staining showed that the injured bile duct healed well, and the bile duct complex gradually degenerated with the extension of implantation time. Immunohistochemical staining and Western blotting showed that the expression levels of epithelial cell markers CK19 and E-cadherin in the *in vivo* complex group were significantly increased compared with the control group [12,40,54]. Over time, bone marrow MSCS can penetrate the material's pores and grow. SIS has good cytocompatibility and is an ideal biological scaffold for bone marrow mesenchymal stem cells [49,55].

In this study, the SIS compound between umbilical cord mesenchymal stem cells to treat full-thickness skin wound injury in rats through the comparison between groups skin wound healing and quality, paraffin section, dyeing, microvascular density observation, the collagen of the wound Masson staining, evaluation between umbilical cord mesenchymal stem cells in a composite SIS the influence of the treatment of full-thickness skin damage wound healing, To provide a new treatment strategy for full-thickness skin lesions.

## Conclusion

In conclusion, the present study showed that porcine small intestinal submucosa had poor efficacy in repairing full-thickness skin injury wounds in rats due to the lack of mesenchymal stem cell support and functional motor cells. Although simple mesenchymal stem cells can repair skin wounds and play a certain repair effect, the stem cell suspension is easy to flow and has a short residence time in the wound, which cannot give full play to the function of stem cells to promote vascular endothelial cells and secrete vascular cytokines within the effective time, thus affecting the effect of cell therapy. Compared with the other three groups, the wound healing speed of human umbilical cord mesenchymal stem cells combined with the porcine small intestinal submucosa group was faster and higher in quality, and the wound was healed into normal skin tissue. The positive rate of CD31 cells in the microvascular count was higher, the skin appendages grew, and the collagen fibers were dense and orderly arranged.

Human umbilical cord mesenchymal stem cells combined with SIS were used to treat full-thickness skin injury. The functional seed cells expanded *in vitro*, were planted on degradable scaffold material, and transplanted to the injured site *in vivo*. Functional seed cells proliferate and differentiate into active tissues with special functions similar to natural tissues and organs in morphology, structure and function. Applying seed cells to promote wound repair can speed healing, repair deep tissues and better treat tissue damage. Its development is expected to solve the problem that cell therapy is not ideal for repairing full-thickness skin wounds. The results suggest that SIS combined with human umbilical cord mesenchymal stem cells can promote angiogenesis and wound repair. It is a beneficial cell therapy to give stem cells as human umbilical cord mesenchymal stem cells and allow the cells to migrate to the damaged area alone. Stenting umbilical cord mesenchymal stem cells may be a safer and more effective treatment for various systemic diseases. However, the characterization of active factors involved in wound healing and paracrine function of human umbilical cord MSCS is at an early stage due to the low sensitivity of the analysis of these factors. Therefore, our next research goal will be determining the active factors of human umbilical cord mesenchymal stem cells. These possible potential factors will profoundly influence the future strategy of regenerative medicine.

In our future research endeavors, we aim to conduct additional experiments to explore various aspects of MSCs. Specifically, we will investigate the synergistic effects of MSCs to enhance proliferation, delve into the multiple differentiation potential of MSCs, and explore the research landscape surrounding MSC composite materials. Expanding the scope of our work to encompass these specific experiments necessitates the allocation of additional resources and time. We are committed to dedicating the necessary resources and time to our future studies to thoroughly investigate and contribute to the understanding of these crucial aspects of MSCs.

**Summary points**

- Full-thickness skin injuries, whether from burns or chronic wounds, pose substantial health risks, including pain, dehydration and bacterial infections, necessitating effective wound treatment strategies.
- Cell therapy, particularly involving mesenchymal stem cells, offers a promising avenue for tissue regeneration, accelerating angiogenesis and supporting tissue healing.
- Bone marrow mesenchymal stem cells play a vital role in wound healing, promoting angiogenesis through the secretion of VEGF A and other critical factors.
- Traditional stem cell delivery methods, such as injections, face challenges due to poor cell retention and limited control over cell placement.
- Developing tissue scaffold materials is essential to provide a 3D scaffold for tissue cells, facilitating cell adhesion, proliferation and differentiation.
- Synthetic biomaterials have limitations, including a lack of biological activity, poor surface hydrophobicity and suboptimal cell adhesion.
- Porcine small intestinal submucosa (SIS) presents a promising alternative as an acellular matrix derived from the small intestine. It comprises a variety of cytokines and provides a conducive environment for cell proliferation, blood vessel formation and differentiation.
- SIS is widely employed in clinical practice for tissue remodeling due to its porous 3D structure, biocompatibility and ability to support cell adhesion, proliferation and differentiation.
- The study introduces a novel approach combining human umbilical cord mesenchymal stem cells with SIS to treat full-thickness skin injuries in rats.
- Results demonstrate that this combination significantly accelerates wound healing and improves wound quality. CD31 staining reveals increased microvessel density and collagen fiber staining shows well-organized collagen fibers.
- SIS combined with human umbilical cord mesenchymal stem cells provides a 3D morphological structure for repairing full-thickness skin injuries, making it a potential solution for addressing the limitations of conventional cell therapy.
- This research suggests that when combined with human umbilical cord mesenchymal stem cells, SIS can effectively promote angiogenesis and wound repair. This approach can become a safer and more effective treatment strategy for systemic diseases.
- The next research goal is to investigate further the active factors involved in wound healing and the paracrine function of human umbilical cord mesenchymal stem cells, which could significantly impact the field of regenerative medicine.
- In summary, this study underscores the potential of combining human umbilical cord mesenchymal stem cells with SIS as a novel and effective strategy for accelerating wound healing, promoting angiogenesis and providing a more reliable solution for various systemic diseases.
